# Incidence, Clinical Features, and Prognostic Value of New‐Onset Renal Impairment in Multiple Myeloma

**DOI:** 10.1002/cam4.71361

**Published:** 2025-11-05

**Authors:** Xiang Liu, Qian Hu, Yuhuan Zheng, Wenjiao Tang, Ting Niu

**Affiliations:** ^1^ Department of Hematology/Institute of Hematology Research West China Hospital, Sichuan University Chengdu China; ^2^ State Key Laboratory of Biotherapy, Collaborative Innovation Center of Biotherapy West China Hospital, Sichuan University Chengdu China; ^3^ National Facility for Translational Medicine (Sichuan), West China Hospital, Sichuan University Chengdu China

**Keywords:** MMRF‐CoMMpass dataset, multiple myeloma, new‐onset renal impairment, risk factor

## Abstract

**Background:**

Previous studies mainly focused on renal impairment (RI) at multiple myeloma (MM) diagnosis, with few investigating new‐onset RI post‐MM diagnosis. This study aims to indicate the incidence, clinical characteristics and predictive value of new‐onset RI in MM patients.

**Methods:**

We conducted a multicenter, retrospective cohort study including 1953 newly diagnosed MM patients from West China Hospital from July 1, 2008, to February 30, 2024 and the international MMRF‐CoMMpass database. Among them, 1770 patients received novel therapeutic agents including immunomodulatory drugs (IMiDs) and proteasome inhibitors (PIs) as first‐line therapy. RI was defined as serum creatinine higher than 2 mg/dL or impaired creatinine clearance (< 40 mL/min/1.73m^2^). The association between new‐onset RI and mortality risk was investigated by Kaplan–Meier analysis and Cox proportional hazard models.

**Results:**

Of the cohort, 16.6% developed new‐onset RI, with the majority (67.1%) occurring within 2 years post‐MM diagnosis. The median overall survival (OS) was significantly shorter in new‐onset RI than in those without RI (68 vs. 122 months, *p* < 0.001). New‐onset RI was an independent risk factor for mortality (HR 1.55, 95% CI 1.28–1.88, *p* < 0.001), and earlier onset of RI was associated with a higher mortality risk. Moreover, patients with recovery of renal function had prolonged OS compared to those without recovery (95 vs. 64.8 months, *p* = 0.01). Older age, higher stage of the international stage system (ISS) and RI at diagnosis seemed to be risk factors for new‐onset RI, while first‐line therapy with PIs and IMiDs combinations was associated with a lower risk of RI development (HR 0.69, 95% CI 0.51–0.94, *p* = 0.017).

**Conclusions:**

In conclusion, the incidence of new‐onset RI is high in MM, and is a significant risk factor for mortality, posing a substantial threat to MM patients. Early identification of high‐risk patients for new‐onset RI and prompt preventive strategies is critical for improving MM prognosis.

**Trial Registration:**

This study was registered by the Chinese Clinical Trial Registry (ChiCTR2400081476, https://www.chictr.org.cn)

## 1 Introduction

Multiple myeloma (MM) accounts for approximately 10% of hematologic malignancies, with a rising global incidence due to population growth, aging, and improved detection, presenting a significant socioeconomic burden [1, 2, 3]. Renal impairment (RI), a prevalent complication of symptomatic MM, affects 20%–50% of patients at diagnosis and is significantly associated with inferior survival in MM [3, 4].

In the past decade, novel therapeutic agents such as immunomodulatory drugs (IMiDs), proteasome inhibitors (PIs), and CD38‐targeting antibodies etc. have significantly improved MM prognosis, and the risk of severe RI in MM has also decreased [5]. However, owing to the ongoing aging of the population, the proportion of patients presenting with RI still remains high [6]. RI continues to pose a significant challenge in the management of MM, particularly in patients who fail treatment, as it is associated with a worsened prognosis and an increased risk of early mortality [7, 8, 9].

Numerous studies have investigated RI in MM patients, including evaluating the change of renal function longitudinally from baseline to 12 months [10], investigating the incidence and outcomes of patients with RI at MM diagnosis [4, 11, 12, 13], as well as examining the factors associated with deterioration or recovery of renal function and the consequent prognostic implications [14, 15]. Nevertheless, very few studies specifically address new‐onset RI after MM diagnosis. The incidence, clinical characteristics and predictive value of the new‐onset RI for the prognosis of MM patients are still largely unknown. To bridge this gap, this study analyzed data from our institutional database and the international multicenter MMRF‐CoMMpass dataset, hoping to provide a deeper understanding of new‐onset RI in MM patients.

## 2 Methods

### 2.1 Ethics

The study was deemed exempt and granted a waiver of consent by the Institutional Review Board of West China Hospital, Sichuan University (approval number: 1783), and was registered by the Chinese Clinical Trial Registry (ChiCTR2400081476, https://www.chictr.org.cn).

### 2.2 Study Design and Participants

This study included patients diagnosed as MM in West China Hospital (China) from July 1, 2008, to Feb 30, 2024, or patients enrolled in the prospective observational Multiple Myeloma Research Foundation (MMRF) CoMMpass study (NCT01454297) which includes data from multi‐centers across Europe and North America. (https://portal.gdc.cancer.gov/projects/MMRF‐COMMPASS).

Patients who (1) were newly diagnosed MM (NDMM) according to International Myeloma Working Group (IMWG) criteria [2], (2) had a baseline creatinine measurement and (3) had at least three creatinine follow‐ups with an interval > 1 month were eligible for inclusion. The exclusion criteria included patients with (1) missing baseline data such as baseline creatinine, ISS stage or first‐line treatment regimen, (2) a follow‐up duration of less than 3 months, and (3) persistent RI from baseline. Guided by the IMWG guidelines, the treatment pathway was individualized based on the patient's performance status, clinical judgment, and participant preference [16].

Clinical and laboratory data at diagnosis were collected, including age, race, gender, ISS stage, platelet count (PLT), white blood cell count (WBC), total protein (TP), albumin (ALB), lactate dehydrogenase (LDH), urea, creatinine, estimated glomerular filtration rate (eGFR), calcium, beta‐2 microglobulin (B2M), kappa free light chain (FLC), lambda FLC, the difference between the involved and the uninvolved FLC (dFLC), M‐protein, first‐line induction therapy etc.

### 2.3 Study Outcomes and Follow Up

Follow‐up data including survival status and creatinine levels were collected via practitioner interview or by reviewing electronic medical records in West China Hospital; all patients were followed until death, last contact or the end date of follow‐up on September 30, 2024. For the MMRF dataset, these follow‐up data were available at https://portal.gdc.cancer.gov/projects/MMRF‐COMMPASS. The primary outcome of this study was all‐cause mortality.

### 2.4 Definitions

MM was diagnosed according to IMWG criteria [2]. RI was defined as serum creatinine higher than 2 mg/dL or impaired creatinine clearance (< 40 mL/min/1.73 m^2^) according to IMWG [16]. New‐onset RI was defined as newly developed RI occurring at least 3 months after MM diagnosis. RI remission was determined as the eGFR improved to ≥ 40 mL/min/1.73m^2^ after treatment [15]. Persistent RI from baseline was defined as patients accompanied by RI at MM diagnosis, and who did not experience RI remission during follow‐up time. The International Staging System (ISS) for MM is defined as follows: Stage I includes patients with serum beta‐2 microglobulin < 3.5 mg/L and serum albumin ≥ 3.5 g/dL; Stage II applies to those who do not meet the criteria for Stage I or Stage III; and Stage III is characterized by serum beta‐2 microglobulin ≥ 5.5 mg/L [17]. The Chronic Kidney Disease Epidemiology Collaboration (CKD‐EPI) equation using creatinine measurements was utilized to estimate the eGFR [18].

### 2.5 Statistical Analysis

Normally distributed data and continuous skewed variables were presented as mean and standard deviation (SD) or median and interquartile ranges (IQRs), respectively. The one‐way ANOVA analysis and the Wilcoxon–Mann–Whitney rank‐sum tests were used to analyze normally distributed data and continuous skewed variables, respectively. Categorical variables are expressed as frequencies (*n*) and percentages (%), and were compared via Chi‐square or Fisher's exact test. The Kaplan–Meier (KM) analyses were performed to compare survival outcomes. In univariate analyses, significant factors related to survival (*p* < 0.05) and variables clinically considered to be closely associated with survival were included in the multi‐variate Cox proportional hazards model. The association between the new‐onset time of RI and mortality risk was evaluated via restricted cubic splines (RCS) (knots at the 10th, 50th and 90th percentiles).

To balance the baseline characteristics between the two groups, we carried out propensity score matching (PSM). PSM was performed to control for confounders in both groups by including age, gender ISS stage, M protein, total protein, LDH, platelet, albumin, urea, creatinine, eGFR, calcium, B2M, dFLC and first‐line therapy as covariates with a caliper size of 0.02.

The R packages of “tableone”, “survival”, “survminer”, “MatchIt”, “rms”, “autoReg” and “ggplot2” were used for standardized difference analysis, survival analysis, PSM and visualization. Two‐sided *p* values < 0.05 were considered statistically significant.

## 3 Results

### 3.1 Baseline Characteristics of New‐Onset RI in MM Patients

**FIGURE 1:**
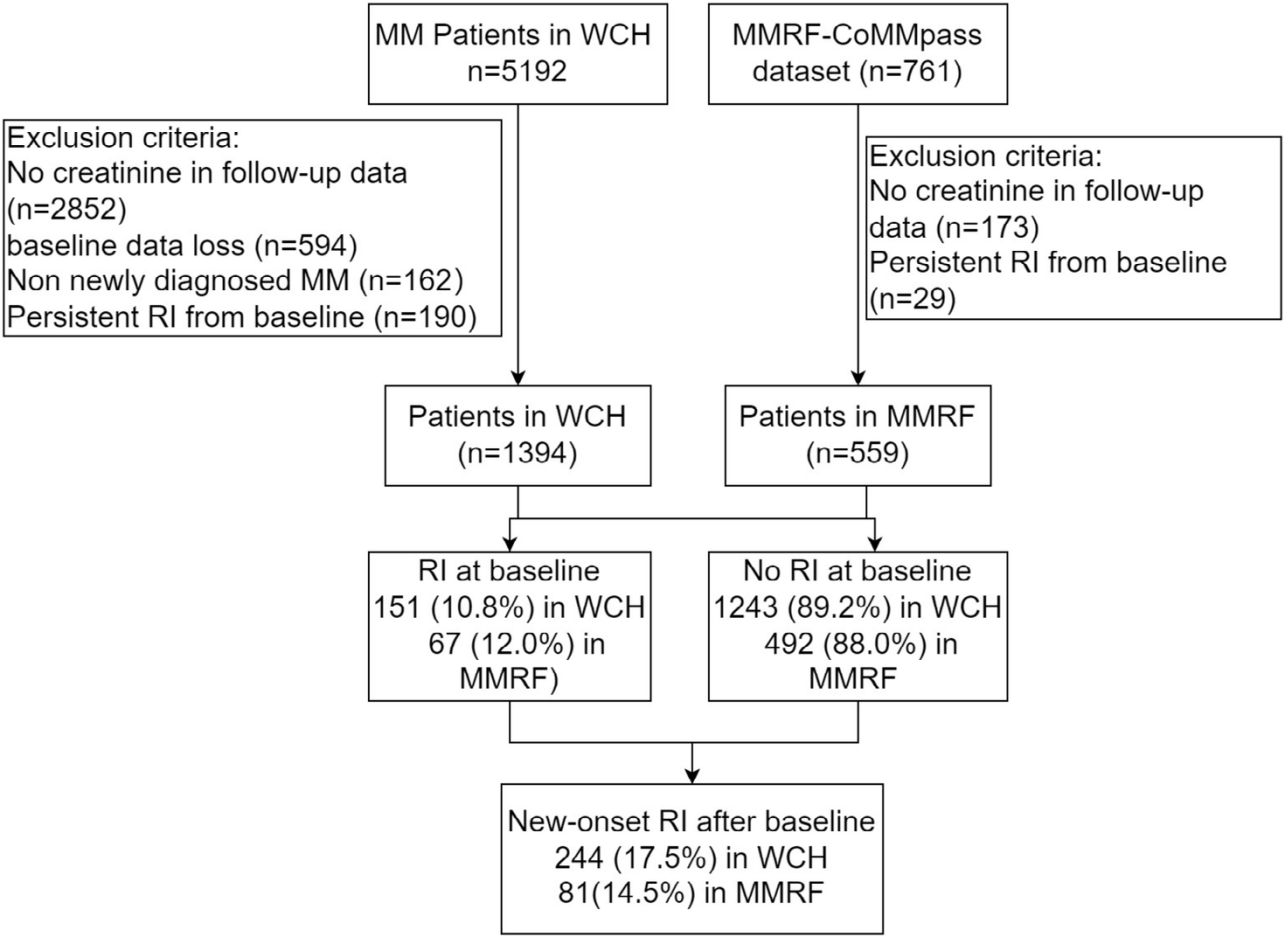
Flow chart of participant enrollment. WCH, West China Hospital; RI, renal impairment.

In West China Hospital (WCH) database, a total of 5192 patients were diagnosed with MM and had available baseline creatinine measurement from July 1, 2008 to February 30, 2024. While in MMRF‐ CoMMpass dataset, a total of 761 MM patients had accessible data at https://portal.gdc.cancer.gov/projects/MMRF‐COMMPASS. Based on the patient selection criteria, 1394 patients from WCH and 559 patients from the CoMMpass dataset were included in the study (Figure 1). All MM patients in the WCH cohort were Asian, whereas the majority in the MMRF‐CoMMpass dataset were of White or Black race (Table S1). As expected, there were significant differences in age, gender, ISS stage and baseline biochemical parameters between the WCH and the MMRF‐CoMMpass cohorts (Table S1).

Among the included 1953 NDMM patients, 10.8% and 12% were with RI at baseline but experienced RI remission after treatment in the WCH and MMRF‐CoMMpass dataset, respectively. During a median follow‐up time of 39 months, 244 (17.5%) in WCH and 81 (14.5%) patients in the MMRF‐CoMMpass dataset developed new‐onset RI after baseline (Figure 1).

A summary of patients' characteristics was provided in Table 1. Compared to MM patients without new‐onset RI, those with new‐onset RI seemed to be older, had a higher ISS stage, a higher level of LDH, urea, creatinine, eGFR, β2M, calcium, dFLC and a lower level of platelet and albumin (Table 1). Notably, those who experienced RI at baseline were prone to develop RI again after MM diagnosis compared with patients without RI at baseline (40.4% vs. 13.7%, *p* < 0.001, Table 1). Additionally, the patients treated with the PI +IMiDs regimen at first‐line therapy were less likely to experience new‐onset RI (*p* < 0.001) (Table 1). There were no differences in gender, race, baseline M protein between the two groups.

**TABLE 1:** The baseline characteristics between the two groups.

Variables	Overall (*n* = 1953)	Non‐new RI (*n* = 1628)	New‐onset RI (*n* = 325)	*p*
Age ≥ 65 years	764 (39.1)	588 (36.1)	176 (54.2)	< 0.001
Race (*n*, %)				0.266
White	433 (20.4)	367 (22.5)	66 (20.3)	
Asian	1404 (71.9)	1159 (71.2)	245 (75.4)	
Black	83 (4.2)	72 (4.4)	11 (3.4)	
Unknown	33 (1.7)	30 (1.8)	3 (0.9)	
Gender, Male (*n*, %)	1049 (53.7)	867 (53.3)	182 (56.0)	0.365
ISS (*n*, %)				< 0.001
I	783 (40.1)	720 (44.2)	63 (19.4)	
II	661 (33.8)	545 (33.5)	116 (35.7)	
III	509 (26.1)	363 (22.3)	146 (44.9)	
Platelet (× 10^9^/L)	179 [131, 232]	182 [135, 235]	168 [112, 217.5]	< 0.001
WBC (× 10^9^/L)	5.60 [4.28, 7.30]	5.60 [4.24, 7.26]	5.60 [4.41, 7.36]	0.391
TP (g/dL)	68.5 [11.4, 87.9]	68.6 [11.2, 88.0]	67.5 [25.5, 87.7]	0.543
Albumin (g/L)	37.60 [32.00, 42.40]	38.0 [32.2, 42.8]	36.1 [30.6, 40.5]	< 0.001
LDH (IU/L)	174.03 [139.99, 219.00]	173.0 [139.0, 215.0]	179.0 [148.0, 238.0]	0.006
Urea (mmol/L)	6.00 [4.70, 7.50]	5.71 [4.64, 7.24]	6.91 [5.50, 9.34]	< 0.001
Creatinine (μmol/L)	77.5 [63.00, 99.00]	75.00 [62.00, 93.00]	95.50 [72.20, 133.84]	< 0.001
Baseline RI	218 (11.2)	130 (8.0)	88 (27.1)	< 0.001
Calcium (mmol/L)	2.33 [2.20, 2.49]	2.32 [2.20, 2.48]	2.35 [2.21, 2.54]	0.047
B2M (mg/L)	3.42 [2.44, 5.65]	3.22 [2.35, 5.08]	5.14 [3.47, 7.74]	< 0.001
Baseline dFLC (mg/L)	118.10 [18.05, 657.55]	102.75 [16.52, 565.10]	255.60 [35.15, 1022.03]	< 0.001
M protein (g/L)	20.59 [4.60, 41.47]	21.41 [4.83, 41.97]	16.02 [3.70, 37.92]	0.067
First‐line induction (*n*, %)				< 0.001
Conventional therapy	183 (9.4)	123 (7.6)	60 (18.5)	
PIs or IMiDs	1225 (62.7)	1013 (62.2)	212 (65.2)	
PIs + IMiDs	545 (27.9)	492 (30.2)	53 (16.3)	
Follow up time (months)	39 [25, 38]	39.2 [25, 58]	38 [22.1, 59.3]	0.579

Abbreviations: B2M, β2‐microglobulin; dFLC, the difference between the involved and the uninvolved free light chain serum levels; IMiDs, immunomodulatory drug; ISS, International Staging System; LDH, lactate dehydrogenase; PIs, proteasome inhibitor; PLT, platelet count; RI, renal impairment; TP, total protein; WBC, white blood cell count.

**FIGURE 2:**
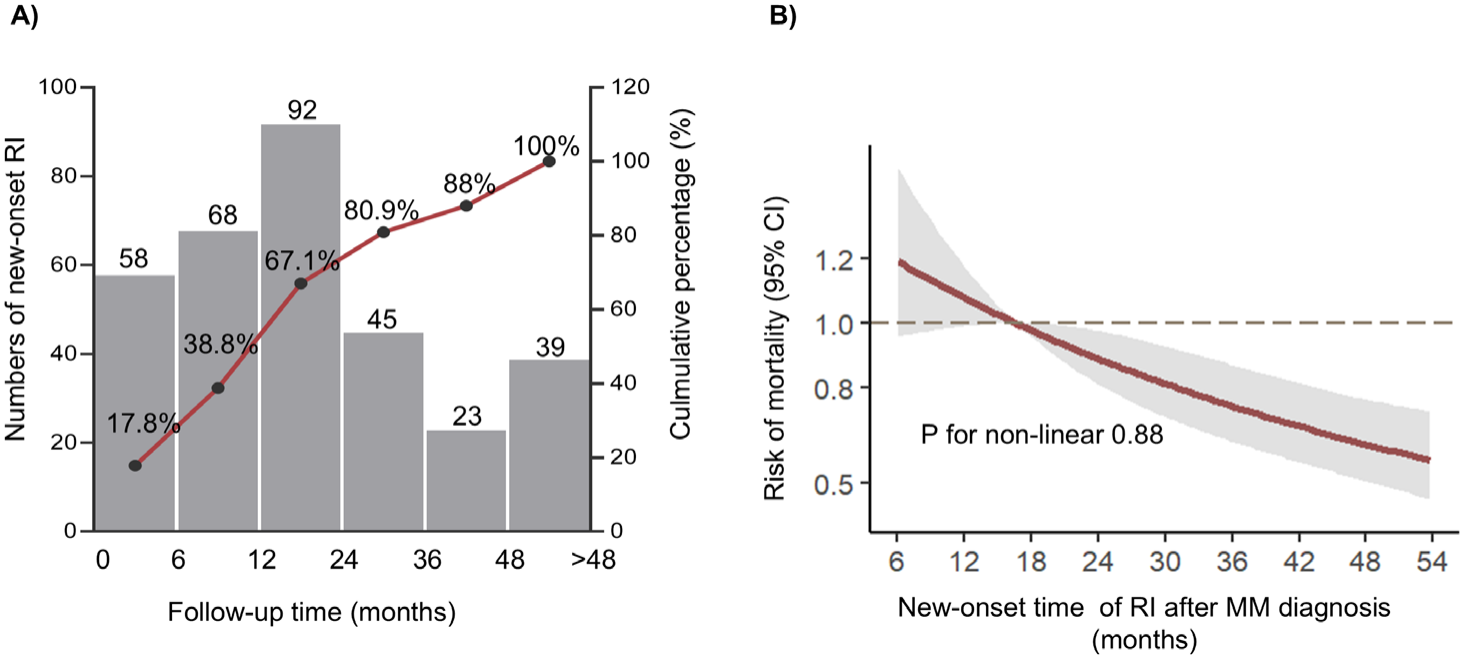
Clinical characteristics of MM patients with new‐onset RI. (A) Histogram showing the frequency of new‐onset RI at different time points after MM diagnosis, with a line graph indicating the cumulative proportion of patients with new‐onset RI. (B) The relationship between the time to new‐onset RI and mortality risk in MM patients by restricted cubic splines curve.

For those new‐onset RI patients, over half of them (67.1%) developed RI within 2 years after MM diagnosis (Figure 2A). The RCS curve revealed that the earlier the onset of RI in MM patients, the higher the patients' mortality risk (*p* for nonlinearity = 0.88, Figure 2B).

### 3.2 New‐Onset RI Is an Independent Risk Factor to All‐Cause Mortality in MM Patients

**FIGURE 3:**
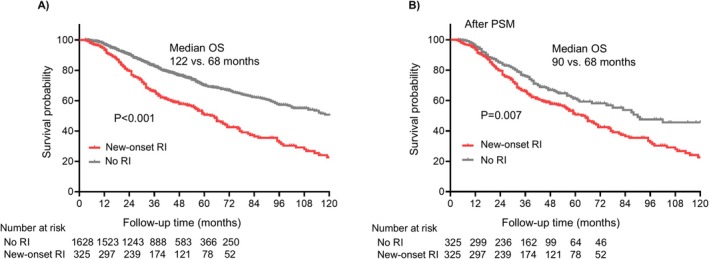
Survival analysis of MM patients between new‐onset RI and no RI group. The comparison of OS in (A) overall patients and (B) patients after PSM. RI, renal impairment. OS, overall survival. PSM, propensity score‐matched.

The mortality risk was significantly higher in the new‐onset RI group (52.3%, 170/325) compared to the non‐new RI group (25.4%, 414/1628). KM survival analysis revealed that the median overall survival (OS) of MM patients with new‐onset RI was 64.8 months (95% CI 56.3–73.3), which was significantly shorter than those without new‐onset RI (median OS 122 months, 95% CI 106.3–137.7) (*p* < 0.001) (Figure 3A). After a 1:1 ratio PSM, a cohort of patients with balanced baseline demographic and clinical characteristics was established (*n* = 325 for each group, Table S2). The median OS for MM patients with new‐onset RI (median OS 64.8 months, 95% CI 56.3–73.3) remained significantly shorter than those without new‐onset RI (median OS 90 months, 95% CI 58.2–121.8) (*p* < 0.001) (Figure 3B). Among the 325 new‐onset RI patients, 214 (65.5%) had following creatinine monitoring, in which 51.6% recovered renal function. KM analysis demonstrated a longer survival for the RI remission group compared to the persistent RI group (median OS 95 vs. 64.8 months, *p* = 0.01, Figure S1).

In the multivariate Cox regression analysis, new‐onset RI was shown to be a significant adverse prognostic factor for mortality (HR 1.55, 95% CI: 1.28–1.88, *p* < 0.001) after adjustment for age, gender, ISS stage, platelet, LDH, baseline creatinine, urea, dFLC and first‐line therapy (Table 2). After PSM, new‐onset RI remained an independent risk factor for all‐cause mortality (HR 1.49, 95% CI: 1.17–1.91, *p* = 0.001, Table S3) in the multivariate COX regression analysis.

**TABLE 2:** The association of new‐onset renal impairment with mortality in patients with multiple myeloma by univariate and multivariate Cox regression analysis.

Variables	Univariate analysis	Multivariate analysis
	Hazard ratio (95% CI, *p*)	Hazard ratio (95% CI, *p*)
New‐onset RI	2.07 (1.73–2.48, *p* < 0.001)	1.55 (1.28–1.88, *p* < 0.001)
Age ≥ 65 years	1.48 (1.25–1.74, *p* < 0.001)	1.30 (1.09–1.54, *p* = 0.003)
Gender (male)	1.27 (1.08–1.50, *p* = 0.004)	1.18 (0.99–1.40, *p* = 0.052)
ISS stage		
ISS I	References	References
ISS II	1.57 (1.27–1.93, *p* < 0.001)	1.34 (1.06–1.71, *p* = 0.014)
ISS III	2.73 (2.23–3.35, *p* < 0.001)	2.10 (1.64–2.69, *p* < 0.001)
PLT (× 10^9^/L)	0.997 (0.996–0.998, *p* < 0.001)	0.998 (0.997–0.999, *p* = 0.004)
LDH (IU/L)	1.00 (1.00–1.00, *p* < 0.001)	1.00 (1.00–1.00, *p* < 0.001)
Creatinine	1.001 (1.000–1.002, *p* = 0.001)	1.00 (0.99–1.00, *p* = 0.706)
Urea	1.04 (1.02–1.05, *p* < 0.001)	0.99 (0.96, 1.01, *p* = 0.304)
Baseline dFLC (mg/mL)	1.00 (1.00–1.00, *p* = 0.028)	1.00 (1.00–1.00, *p* = 0.775)
First‐line therapy		
PIs or IMiDs	References	References
Conventional therapy	1.52 (1.22–1.88, *p* < 0.001)	1.38 (1.11–1.72, *p* = 0.004)
PIs combined with IMiDs	0.75 (0.60–0.94, *p* = 0.011)	0.84 (0.66–1.07, *p* = 0.151)

Abbreviations: dFLC, the difference between the involved and the uninvolved free light chain serum levels; IMiDs, immunomodulatory drug; ISS, international staging system; LDH, lactate dehydrogenase; PIs, proteasome inhibitor; PLT, platelet count; RI, renal impairment.

Next, we performed a stratified analysis to further investigate the predictive impact of new‐onset RI on MM prognosis. The results indicated that new‐onset RI emerged as a significant independent prognostic factor across all subgroups of MM patients, including age ≥ 65 years, ISS stage (I, II or III), race (White or Asian) and the type of first‐line therapy (including PIs or IMiDs and PIs+IMiDs) (Table S4). Furthermore, we also investigated the association between new‐onset RI and survival through separate analyses of the WCH and MMRF patient cohorts. As shown in Figure S2, the survival of patients with new‐onset RI was shorter than that of the no RI group in both cohorts (*p* < 0.001), and in multivariate COX regression analysis, new‐onset RI was indicated to be a significant risk factor for mortality both in patients from the WCH and MMRF databases (Table S5).

### 3.3 Risk Factors for New‐Onset RI in MM

To investigate risk factors for new‐onset RI in MM patients, we performed univariate and multivariate Cox regression analyses. The results identified baseline RI (HR 2.44, 95% CI 1.84–3.25, *p* < 0.001), ISS stage II (HR 2.14, 95% CI 1.56–2.92, *p* < 0.001) and stage III (HR 3.02, 95% CI 2.16–4.21, *p* < 0.001) as the most significant risk factors for new‐onset RI, followed by age ≥ 65 years (HR 2.12, 95% CI 1.69–2.67, *p* < 0.001) and LDH (HR 1.001, 95% CI 1.000–1.001, *p* < 0.001) (Table 3). Conversely, MM patients with PIs combined with IMiDs therapy at first line were associated with a lower risk of new‐onset RI (HR 0.69, 95% CI 0.51–0.94, *p* = 0.017) compared to those with only PIs or IMiDs therapy at first line (Table 3). These findings highlight the critical role of age, ISS stage, baseline renal function, and first‐line induction therapy in the development of new‐onset RI.

**TABLE 3:** Univariate and multivariate Cox regression analysis of factors predicting new‐onset renal impairment in multiple myeloma patients.

Variables	Univariate analysis	Multivariate analysis
	Hazard ratio (95% CI, *p*)	Hazard ratio (95% CI, *p*)
Age ≥ 65 years	2.29 (1.84–2.85, *p* < 0.001)	2.12 (1.69–2.67, *p* < 0.001)
ISS stage		
ISS I	Reference	Reference
ISS II	2.39 (1.76–3.25, *p* < 0.001)	2.14 (1.56–2.92, *p* < 0.001)
ISS III	4.79 (3.56–6.44, *p* < 0.001)	3.02 (2.16–4.21, *p* < 0.001)
PLT (× 109/L)	0.997 (0.996–0.999, *p* < 0.001)	0.999 (0.998–1.001, *p* = 0.282)
LDH (IU/L)	1.001 (1.000–1.001, *p* < 0.001)	1.001 (1.000–1.001, *p* < 0.001)
Adjusted calcium	1.43 (1.15–1.79, *p* = 0.002)	0.97 (0.73–1.27, *p* = 0.804)
Baseline RI	4.09 (3.20–5.23, *p* < 0.001)	2.44 (1.84–3.25, *p* < 0.001)
Baseline dFLC (mg/mL)	1.00 (1.00–1.00, *p* < 0.001)	1.00 (1.00–1.00, *p* = 0.174)
First‐line induction		
PIs or IMiDs	Reference	Reference
Conventional therapy	1.85 (1.39–2.47, *p* < 0.001)	2.16 (1.60–2.90, *p* < 0.001)
PIs + IMiDs combination	0.58 (0.43–0.78, *p* < 0.001)	0.69 (0.51–0.94, *p* = 0.017)

Abbreviations: dFLC, the difference between the involved and the uninvolved free light chain serum levels; IMiDs, immunomodulatory drug; ISS, international staging system; LDH, lactate dehydrogenase; PIs, proteasome inhibitor; PLT, platelet count; RI, renal impairment.

## 4 Discussion

In this international, multicenter cohort study, we present the first comprehensive analysis of the incidence, clinical characteristics, and prognostic significance of new‐onset RI in MM patients. Our data demonstrate a 16.6% incidence of new‐onset RI, with the highest risk occurring within the first 2 years post‐MM diagnosis. Notably, MM patients developing RI exhibited significantly elevated mortality risk, and earlier RI onset correlated with worse survival outcomes. Multivariate analysis identified age, advanced ISS stage and baseline RI as independent risk factors for new‐onset RI, while PIs combined with IMiDs treatment emerged as a protective factor.

MM is considered the most common malignancy that leads to RI [19]. If the injured kidney is not treated, there is rapid progression of glomerular or tubular injury to non‐reversible nephron fibrosis and irreversible renal failure, finally resulting in slowly progressive chronic kidney disease and, in severe cases, advancing to end‐stage renal disease (ESRD) requiring dialysis [20, 21]. Therefore, we believe that in addition to focusing on the occurrence of RI at the time of MM diagnosis, it is equally important to clarify the incidence and prognosis of new‐onset RI during the course of MM. Here, we observed a 16.6% morbidity of new‐onset RI in MM patients after diagnosis, and new‐onset RI was identified as an independently significant risk factor for mortality in MM patients, with a 55% increased risk of death compared to patients without new‐onset RI.

Interestingly, we found the majority of new‐onset RI patients (67.1%) develop RI within 2 years after MM diagnosis, and the earlier the onset of RI was associated with the higher risk of mortality (Figure 2B), which further validated the vital role of new‐onset RI on the prognosis in MM. On one hand, the presence of RI implies a heavy burden of MM [22, 23], and also poses major management challenges such as optimal antimyeloma therapy, specific non‐pharmacologic therapies (such as dialysis) and eligibility for stem cell transplantation etc., These factors may contribute to the MM progression [24]. On the other hand, MM is considered the most common malignancy that leads to end‐stage kidney disease (ESRD) [25], because only a part of RI patients in MM can recover renal function, while others develop chronic kidney disease, and even progress to ESRD in severe cases, which may significantly increase the mortality risk in MM patients. In this study, we found approximately 49.8% of new‐onset RI patients recovered renal function after treatment; the recovery rate was similar to those who diagnosed RI at MM diagnosis [26]. As expected, renal recovery was accompanied by improved survival (median OS 5 vs. 7.92 years, *p* = 0.003), consistent with previous studies [15]. These findings collectively demonstrate that comprehensive renal management—including early monitoring, prevention of new‐onset RI, and timely intervention—is essential for improving MM patient outcomes.

Previous studies reported that patients with RI at MM diagnosis were older and at a higher ISS stage [15, 27]. Similarly, we found these patients were also prone to develop RI after MM diagnosis (Table 3). With the advancements in drug therapies, many MM patients with RI at diagnosis recover renal function after treatment and have prolonged survival [27, 28]. However, we found these patients were more likely to experience RI again during the following MM progression (HR 2.44, 95% CI 1.84–3.25, *p* < 0.001, Table 2), this may be one of the reasons why MM patients presenting with RI still have a higher mortality risk despite renal function recovery than patients without RI at diagnosis, as reported by many previous studies [11, 15]. Notably, we found the combination of PIs and IMiDs at first‐line therapy could lower the risk of new‐onset RI in MM patients compared to PIs or IMiDs alone. This finding is consistent with many reported research studies, which demonstrated that patients treated with combination therapy including PIs and IMiDs together in early treatment lines were more likely to have a complete renal response (CRR) [26]. At present, newer agents such as daratumumab and pomalidomide have demonstrated favorable efficacy in renal recovery for MM patients with RI, which may also confer benefits in preventing RI occurrence [29, 30].

Our study has several strengths. Firstly, this is a large, international and multi‐center cohort study; our findings are applicable to diverse ethnic groups and multinational populations. Secondly, the large sample size enabled us to conduct a wide range of subgroup and sensitivity analyses, further enhancing the robustness of our results. At last, our research has novelty; we focused on new‐onset RI during MM progression, and firstly reported its clinical characteristics, risk factors, and predictive value for prognosis in MM patients. However, as a retrospective study, our findings are subject to inherent limitations, including confounding factors and selection bias. Besides, since the treatment for MM patients with RI is conventional anti‐MM therapy, lacking specific treatment plans targeting renal impairment, very few patients underwent renal biopsy, leading to the pathological causes of renal injury in MM patients remaining unclear in this study. Lastly, the eGFR was calculated using the CKD‐EPI creatinine equation, as recommended by the IMWG [16]. However, prior studies have suggested that the CKD‐EPI equation incorporating both creatinine and cystatin C offers greater sensitivity for detecting renal impairment and superior prognostic value for overall survival in NDMM [31, 32], combining filtration markers (creatinine and cystatin C) is more accurate and could support better clinical decisions than either marker alone, especially in individuals at risk for or having chronic kidney disease [33]. Therefore, the optimal formula for defining RI in patients with MM remains to be elucidated and warrants further investigation.

In conclusion, new‐onset RI poses a significant threat to MM patients. The early identification of high‐risk patients for new‐onset RI, regular renal function monitoring and timely preventive measures against RI development are critically essential. PI combined with IMiDs therapy is recommended to reduce the risk of new‐onset RI in NDMM.

## Author Contributions

**Xiang Liu:** conceptualization (lead), data curation (equal), funding acquisition (equal), investigation (lead), methodology (equal), resources (equal), software (equal), visualization (equal), writing – original draft (equal), writing – review and editing (equal). **Qian Hu:** data curation (equal), formal analysis (equal), funding acquisition (equal), methodology (equal), software (equal), visualization (equal), writing – original draft (equal). **Yuhuan Zheng:** data curation (equal), formal analysis (equal), resources (equal), writing – review and editing (equal). **Wenjiao Tang:** data curation (equal), formal analysis (equal), resources (equal), validation (equal). **Ting Niu:** conceptualization (equal), funding acquisition (equal), investigation (equal), project administration (equal), writing – review and editing (equal).

## Ethics Statement

The study was approved by the Institutional Review Board of West China Hospital, Sichuan University (approval number: 1783).

## Conflicts of Interest

The authors declare no conflicts of interest.

## Supporting information


Data S1.

## Data Availability

The datasets generated during and/or analyzed during the current study are available from the corresponding author on reasonable request.
