# Correction to “Incidence, Clinical Features, and Prognostic Value of New‐Onset Renal Impairment in Multiple Myeloma”

**DOI:** 10.1002/cam4.71545

**Published:** 2026-02-03

Citation to article being corrected

Xiang, L, Qian, H, Yuhuan, Z et al “Incidence, Clinical Features, and Prognostic Value of New‐Onset Renal Impairment in Multiple Myeloma”, *Cancer Medicine*, 2025;14(21):e71361. https://doi.org/10.1002/cam4.71361

The designation of Citation 1 was incorrect. The correct Citation 1 is “^1^ Department of Hematology, Institute of Hematology, West China Hospital, Sichuan University, Chengdu, China.”

The authors apologize for this error.

