# Supplementary material for: Incidence, Clinical Features, and Prognostic Value of New‐Onset Renal Impairment in Multiple Myeloma
Source: Cancer Med. 2025 Nov 5;14(21):e71361. doi: 10.1002/cam4.71361 (PMC12588725; doi:10.1002/cam4.71361)

**Supplemental Table 1. The comparison of baseline characteristics in MM patients from West China Hospital and CoMMpass dataset.**

| Variables | West China Hospital  (n=1394) | CoMMpass dataset  (n=559) | *P* value |
| --- | --- | --- | --- |
| Age (years) | 61.0 [53.0, 68.0] | 64.0 [58.0, 71.0] | <0.001 |
| Race (n, %) |  |  | <0.001 |
| White | 0 (0) | 433 (77.5) |  |
| Asian | 1394 (100) | 10 (1.8) |  |
| Black | 0 (0) | 83 (14.8) |  |
| Unknown | 0 (0) | 33 (5.9) |  |
| Gender, male (n, %) | 721 (51.7) | 328 (58.7) | 0.005 |
| ISS stage (n, %) |  |  | 0.036 |
| I | 580 (41.6) | 203 (36.3) |  |
| II | 449 (32.2) | 212 (37.9) |  |
| III | 365 (26.2) | 144 (25.8) |  |
| PLT (× 109/L) | 167 [119, 218] | 216 [167, 265] | <0.001 |
| WBC (× 109/L) | 5.4 [4.2, 7.0] | 5.9 [4.5, 7.6] | <0.001 |
| TP (g/dL) | 77.2 [67.0, 98.8] | 90.0 [75.0, 103.0] | <0.001 |
| Albumin (g/L) | 38.3 [32.1, 43.2] | 36.0 [32.0, 40.0] | <0.001 |
| LDH (IU/L) | 176.0 [142.0, 221.0] | 171.0 [136.0, 213.0] | 0.120 |
| Urea (mmol/L) | 5.8 [4.6, 7.3] | 6.4 [5.0, 8.2] | <0.001 |
| Creatinine (µmol/L) | 76.0 [62.0, 97.0] | 81.3 [66.3, 103.4] | <0.001 |
| eGFR (mL/min/1.73m2) | 85.3 [64.3, 98.5] | 81.7 [62.7, 96.3] | 0.067 |
| Calcium (mmol/L) | 2.3 [2.2, 2.4] | 2.5 [2.3, 2.6] | <0.001 |
| β2M (mg/L) | 3.4 [2.4, 5.7] | 3.4 [2.5, 5.6] | 0.849 |
| Baseline dFLC (mg/L) | 68.2 [11.7, 342.2] | 497.9 [88.6, 1848.7] | <0.001 |
| M protein (g/L) | 16.2 [3.7, 40.8] | 29.0 [12.0, 43.4] | <0.001 |
| First-line therapy |  |  |  |
| Conventional | 183 (13.1) | 0 (0) |  |
| PIs or IMiDs | 967 (69.4) | 258 (46.2) |  |
| PIs+IMiDs | 244 (17.5) | 301 (53.8) |  |

Abbreviations: PLT, platelet count. WBC, white blood cell count. TP, total protein. LDH, lactate dehydrogenase. eGFR, estimated glomerular filtration rate. B2M, β2-microglobulin. dFLC, the difference between the involved and the uninvolved free light chain serum levels. PIs, proteasome inhibitor. IMiDs, immunomodulatory drug.

**Supplemental Table 2. The baseline characteristics between the two groups after PSM.**

| Variables | Overall (n=650) | Non-new RI (n=325) | New-onset RI (n=325) | *P* value |
| --- | --- | --- | --- | --- |
| Age (years) | 66.0 [59.0, 73.0] | 66.0 [59.0, 72.0] | 66.0 [58.0, 73.0] | 0.817 |
| Race (n, %) |  |  |  | 0.615 |
| White | 140 (21.5) | 74 (22.8) | 66 (20.3) |  |
| Asian | 478 (73.5) | 233 (71.7) | 245 (75.4) |  |
| Black | 23 (3.5) | 12 (3.7) | 11 (3.4) |  |
| Unknown | 9 (1.4) | 6 (1.8) | 3 (0.9) |  |
| Gender, male (n, %) | 358 (55.1) | 176 (54.2) | 182 (56.0) | 0.693 |
| ISS stage (n, %) |  |  |  |  |
| I | 132 (20.3) | 69 (21.2) | 63 (19.4) | 0.824 |
| II | 227 (34.9) | 111 (34.2) | 116 (35.7) |  |
| III | 291 (44.8) | 145 (44.6) | 146 (44.9) |  |
| PLT (× 109/L) | 166.0 [113.0, 217.7] | 163.0 [116.0, 218.0] | 168.0 [112.0, 217.0] | 0.922 |
| WBC (× 109/L) | 5.6 [4.3, 7.4] | 5.7 [4.1, 7.5] | 5.6 [4.4, 7.4] | 0.668 |
| TP (g/dL) | 67.2 [11.7, 86.6] | 66.9 [11.2, 85.3] | 67.5 [36.6, 87.6] | 0.5 |
| Albumin (g/L) | 36.2 [31.1, 41.2] | 36.5 [31.8, 42.1] | 36.1 [30.8, 40.5] | 0.202 |
| LDH (IU/L) | 181.0 [146.0, 236.0] | 186.0 [143.0, 231.0] | 179.0 [148.0, 238.0] | 0.97 |
| Urea (mmol/L) | 6.9 [5.5, 9.3] | 6.9 [5.6, 9.4] | 6.9 [5.5, 9.3] | 0.922 |
| Creatinine (µmol/L) | 93.0 [72.1, 133.0] | 90.0 [72.0, 132.6] | 95.5 [72.4, 133.5] | 0.528 |
| eGFR (mL/min/1.73m2) | 65.1 [44.9, 86.2] | 66.3 [46.7, 85.8] | 64.1 [40.4, 86.4] | 0.774 |
| Calcium (mmol/L) | 2.4 [2.2, 2.6] | 2.4 [2.2, 2.6] | 2.4 [2.2, 2.5] | 0.986 |
| B2M (mg/L) | 5.1 [3.3, 7.9] | 5.0 [3.0, 8.1] | 5.1 [3.5, 7.7] | 0.276 |
| Baseline dFLC (mg/L) | 194.2 [31.9, 947.9] | 148.2 [26.5, 887.3] | 255.6 [35.2, 1019.9] | 0.084 |
| M protein (g/L) | 17.0 [3.8, 37.9] | 17.2 [3.9, 38.0] | 16.0 [3.8, 37.9] | 0.98 |
| First-line induction (n, %) |  |  |  | 0.5 |
| Conventional therapy | 109 (16.8) | 49 (15.1) | 60 (18.5) |  |
| PIs or IMiDs | 435 (66.9) | 223 (68.6) | 212 (65.2) |  |
| PIs+IMiDs combination | 106 (16.3) | 53 (16.3) | 53 (16.3) |  |

Abbreviations: PSM. propensity score matching. PLT, platelet count. WBC, white blood cell count. TP, total protein. LDH, lactate dehydrogenase. eGFR, estimated glomerular filtration rate. B2M, β2-microglobulin. dFLC, the difference between the involved and the uninvolved free light chain serum levels. PIs, proteasome inhibitor. IMiDs, immunomodulatory drug.

**Supplemental Table 3. The association of new-onset RI with all-cause mortality in multiple myeloma patients by multivariate COX regression after PSM.**

| Variables | Hazard ratio (95% CI) | P |
| --- | --- | --- |
| New-onset RI | 1.49 (1.17-1.91) | 0.001 |
| Age ≥ 65years | 1.13 (0.88-1.45) | 0.326 |
| Gender (male) | 1.21 (0.94-1.55) | 0.137 |
| ISS stage |  |  |
| ISS I | References |  |
| ISS II | 1.62 (1.09-2.42) | 0.018 |
| ISS III | 2.65 (1.80-3.91) | <.001 |
| platelet (× 10^9^/L) | 0.998 (0.997-1.000) | 0.05 |
| Total protein | 0.999(0.995-1.003) | 0.52 |
| LDH (IU/L) | 1.001 (1.000-1.001) | <0.001 |
| Creatinine | 1.00 (0.99-1.00) | 0.543 |
| Urea | 0.98 (0.95, 1.01) | 0.186 |
| Baseline dFLC (mg/mL) | 1.00 (1.00-1.00) | 0.161 |
| First-line induction |  |  |
| PIs or IMiDs | References |  |
| Conventional therapy | 1.28 (0.96-1.69) | 0.09 |
| PIs combined with IMiDs | 0.95 (0.63-1.00) | 0.80 |

Abbreviations: PSM. propensity score matching. RI, renal impairment. ISS, international staging system. LDH, lactate dehydrogenase. PIs, proteasome inhibitor. IMiDs, immunomodulatory drug.

**Supplemental Table 4. The association of new-onset RI with all-cause mortality in different subgroups by multivariate COX regression in MM patients.**

| Variables | HR (95% CI) | P value |
| --- | --- | --- |
| **age≥65** |  |  |
| New-onset RI | 1.39 (1.06-1.82) | 0.019 |
| Gender (male) | 1.02(0.79-1.33) | 0.861 |
| **Age<65** |  |  |
| New-onset RI | 1.91 (1.47-2.48) | <0.001 |
| Gender (male) | 1.35 (1.08-1.70) | 0.009 |
| **ISS I** |  |  |
| New-onset RI | 1.64 (1.04-2.59) | 0.034 |
| Age≥65 | 1.53 (1.09-2.14) | 0.016 |
| Gender (male) | 1.53 (1.09-2.15) | 0.015 |
| **ISS II** |  |  |
| New-onset RI | 1.81 (1.32-2.48) | <0.001 |
| Age≥65 | 1.09 (0.82-1.46) | 0.56 |
| Gender (male) | 1.15 (0.86-1.53) | 0.35 |
| **ISS III** |  |  |
| New-onset RI | 1.50 (1.14-1.99) | 0.004 |
| Age≥65 | 1.32 (1.01-1.73) | 0.045 |
| Gender (male) | 1.16 (0.87-1.54) | 0.306 |
| **White** |  |  |
| New-onset RI | 1.59 (0.99-2.55) | 0.05 |
| Age≥65 | 1.79 (1.16-2.78) | 0.009 |
| Gender (male) | 1.46 (0.94-2.27) | 0.095 |
| **Asian** |  |  |
| New-onset RI | 1.63 (1.31-2.02) | <0.001 |
| Age > 65 | 1.19 (0.98-1.45) | 0.078 |
| Gender (male) | 1.13 (0.93-1.37) | 0.204 |
| **PIs or IMiDs based first-line therapy** |  |  |
| New-onset RI | 1.57 (1.24-1.99) | <.001 |
| Age≥65 | 1.27 (1.03-1.56) | 0.029 |
| Gender (male) | 1.10 (0.88-1.36) | 0.391 |
| **PIs + IMiDs–based first-line therapy** |  |  |
| New-onset RI | 1.99 (1.15-3.46) | 0.015 |
| Age≥65 | 1.40(0.89-2.22) | 0.145 |
| Gender (male) | 2.24(1.41-3.55) | 0.001 |

All models were adjusted for age, gender, ISS stage, platelet, total protein, LDH, creatinine and dFLC values. Abbreviations: RI, renal impairment. ISS, international staging system. LDH, lactate dehydrogenase. PIs, proteasome inhibitor. IMiDs, immunomodulatory drug.

**Supplemental Table 5. The association of new-onset renal impairment with mortality in MM patients from West China Hospital and MMRF database by multivariate Cox regression analysis.**

| Variables | Hazard ratio (95% CI) | |
| --- | --- | --- |
|  | West China Hospital | MMRF dataset |
| New-onset RI | 1.54 (1.24- 1.91, p<0.001) | 1.58 (1.05-2.39, p=0.03) |
| Age ≥ 65years | 1.23 (1.01- 1.49, p=0.042) | 1.55 (1.06-2.26, p=0.023) |
| Gender (male) | 1.11 (0.91- 1.34, p=0.298) | 1.53 (1.04-2.25, p=0.031) |
| ISS stage |  |  |
| ISS I | References | References |
| ISS II | 1.43 (1.13-1.83, p=0.003) | 1.43 (0.89-2.31, p=.142) |
| ISS III | 2.12 (1.64- 2.73, p<0.001) | 2.40 (1.48-3.91, p<0.001) |
| PLT (× 10^9^/L) | 0.99 (0.99- 1.00, p=0.029) | 0.997 (0.994-0.999, p=.008) |
| LDH (IU/L) | 1.00 (1.00- 1.00, p<0.001) | 1.00 (1.00-1.00, p=0.004) |
| Creatinine | 0.37 (0.99- 1.00, p=0.367) | 1.00 (0.99-1.00, p=0.538) |
| Urea | 0.97 (0.93- 1.00, p=0.076) | 1.01 (0.97, 1.06, p=0.600) |
| Baseline dFLC (mg/mL) | 1.00 (1.00-1.00, p=0.061) | 1.00 (1.00-1.00, p=0.649) |
| First-line therapy |  |  |
| PIs or IMiDs | References | References |
| Conventional therapy | 1.43 (1.14- 1.78, p=0.002) | - |
| PIs combined with IMiDs | 0.89 (0.63- 1.27, p=0.546) | 0.80 (0.55-1.15, p=0.225) |

Abbreviations: RI, renal impairment. ISS, international staging system. PLT, platelet count. LDH, lactate dehydrogenase. dFLC, the difference between the involved and the uninvolved free light chain serum levels. PIs, proteasome inhibitor. IMiDs, immunomodulatory drug.

**Supplemental Figure 1. Survival analysis of MM patients between remission and non-remission group in MM patients with new-onset RI.** RI, renal impairment. OS, overall survival.

**
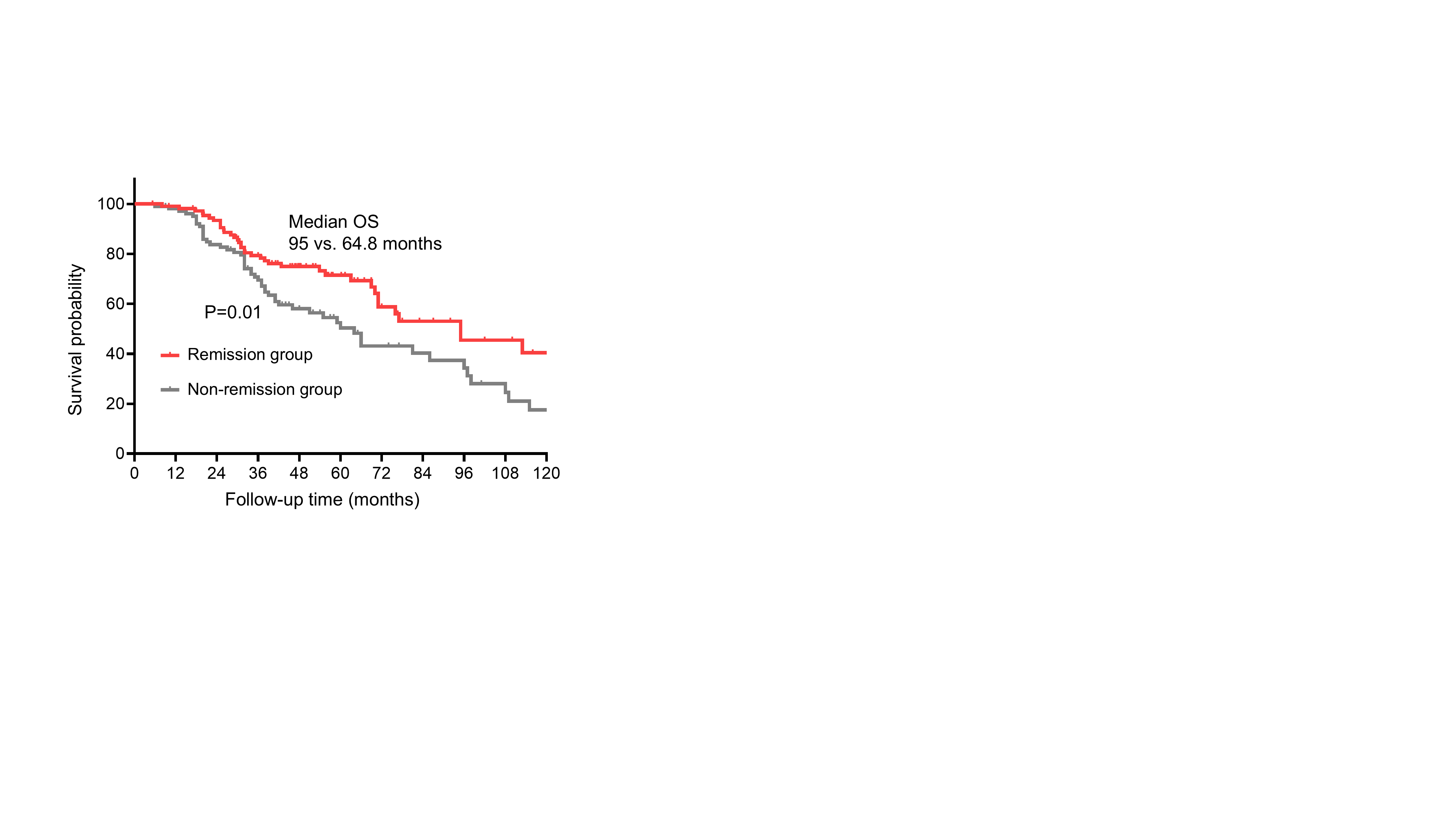
**

**Supplemental Figure 2. Survival analysis of MM patients between new-onset RI and no RI group. The comparison of OS in patients from (A) West China Hospital and (B) MMRF-CoMMpass database.** RI, renal impairment; OS, overall survival.
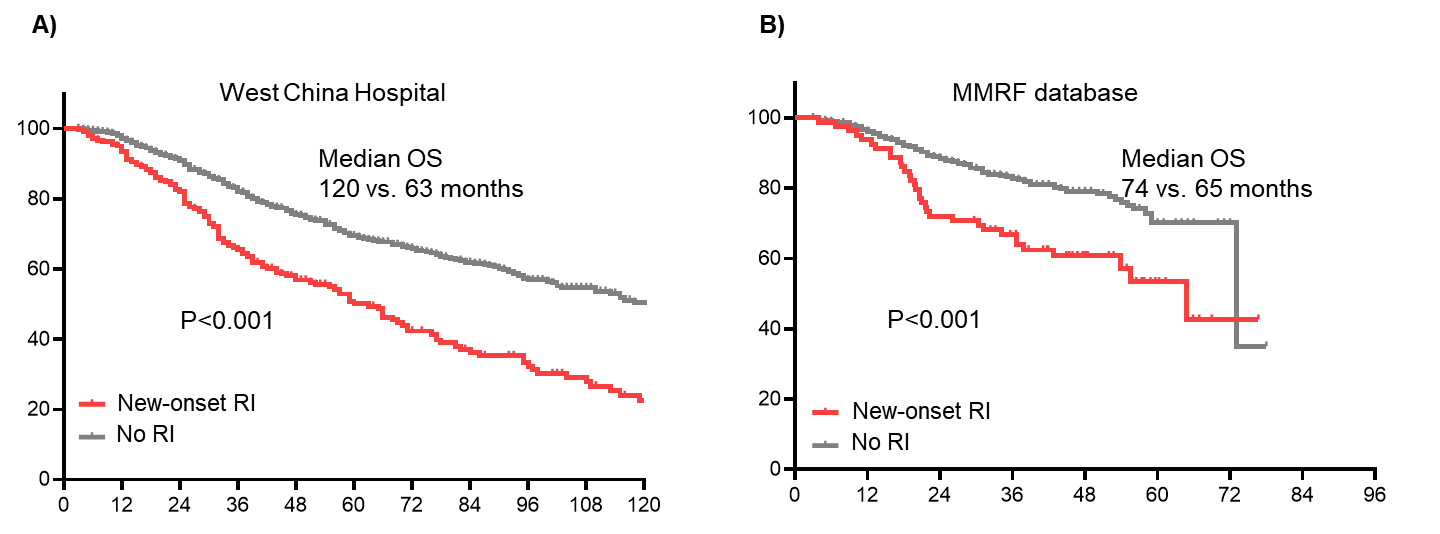

Supplement: Supplementary file 1 — Data S1. [file CAM4-14-e71361-s001.docx]
